# Prognostic values of proliferating cell nuclear antigen (PCNA) and Ki-67 for radiotherapy of oesophageal squamous cell carcinomas

**DOI:** 10.1038/sj.bjc.6690368

**Published:** 1999-05-01

**Authors:** Y Okuno, Y Nishimura, I Kashu, K Ono, M Hiraoka

**Affiliations:** Department of Radiology, Faculty of Medicine, Kyoto University, 54 Shogoin-Kawahara-cho, Sakyo, Kyoto 606-8507, Japan; Department of Radiology, Kinki University School of Medicine, Osaka, 589-8511, Japan; Department of Pathology, Kyoto Katsura Hospital, Yamada-hiraocho, Kyoto 615-8256, Japan; Research Reactor Institute, Kyoto University, Kumatori-cho, Osaka 590-0451, Japan

**Keywords:** PCNA, Ki-67, oesophageal cancer, radiation therapy, local control

## Abstract

The relationship of immunohistochemical indices of proliferating cell nuclear antigen (PCNA) and Ki-67 to local control and survival rates for patients with oesophageal squamous cell carcinomas treated by definitive radiotherapy (RT) was investigated. Biopsy materials before RT were obtained from 65 patients with oesophageal cancer. The median PCNA labelling index (LI) and the median Ki-67 LI were 52% and 45% respectively. The PCNA LI was independent of known prognostic factors on local control for oesophageal cancer, although Ki-67 LI correlated with several prognostic factors. In the univariate analysis, patients with the PCNA LI of < 52% or the Ki-67 LI of < 45% showed significantly higher local recurrence rates than those with higher LIs (both*P* < 0.05). This difference in local control rate according to LIs was prominent for the patients treated with conventional fractionation. In the multivariate analysis, T-stage (*P* = 0.0056) and PCNA LI (*P* = 0.0332) were significant factors for local control in the final model using a stepwise regression procedure. In conclusion, PCNA LI and Ki-67 LI were significantly correlated with local control probabilities in oesophageal squamous cell carcinomas treated by definitive RT. © 1999 Cancer Research Campaign


*British Journal of Cancer* (1999) **80**(3/4), 387–395
© 1999 Cancer Research Campaign
Article no. bjoc.1998.0368

### Prognostic values of proliferating cell nuclear antigen (PCNA) and Ki-67 for radiotherapy of oesophageal squamous cell carcinomas

**Y Okuno[1], Y Nishimura[2], I Kashu[3], K Ono[4] and M Hiraoka[1]**

[1]Department of Radiology, Faculty of Medicine, Kyoto University, 54 Shogoin-Kawahara-cho, Sakyo, Kyoto 606-8507, Japan; [2]Department of Radiology, Kinki University School of Medicine, Osaka, 589-8511, Japan; [3]Department of Pathology, Kyoto Katsura Hospital, Yamada-hiraocho, Kyoto 615-8256, Japan; [4]Research Reactor Institute, Kyoto University, Kumatori-cho, Osaka 590-0451, Japan

**Summary** The relationship of immunohistochemical indices of proliferating cell nuclear antigen (PCNA) and Ki-67 to local control and survival rates for patients with oesophageal squamous cell carcinomas treated by definitive radiotherapy (RT) was investigated. Biopsy materials before RT were obtained from 65 patients with oesophageal cancer. The median PCNA labelling index (LI) and the median Ki-67 LI were 52% and 45% respectively. The PCNA LI was independent of known prognostic factors on local control for oesophageal cancer, although Ki-67 LI correlated with several prognostic factors. In the univariate analysis, patients with the PCNA LI of $< 52\%$ or the Ki-67 LI of $< 45\%$ showed significantly higher local recurrence rates than those with higher LIs (both $P < 0.05$). This difference in local control rate according to LIs was prominent for the patients treated with conventional fractionation. In the multivariate analysis, T-stage ($P = 0.0056$) and PCNA LI ($P = 0.0332$) were significant factors for local control in the final model using a stepwise regression procedure. In conclusion, PCNA LI and Ki-67 LI were significantly correlated with local control probabilities in oesophageal squamous cell carcinomas treated by definitive RT.

**Keywords:** PCNA; Ki-67; oesophageal cancer; radiation therapy; local control

Repopulation of surviving clonogenic tumour cells during the course of fractionated radiotherapy (RT) is a critical factor in determining tumour response and local control (Withers et al, 1988; Trott, 1990). To overcome tumour cell repopulation, a variety of accelerated schedules of RT with a shortened overall treatment time (OTT) were investigated. In our previous studies of oesophageal squamous cell carcinomas, accelerated hyperfractionation (AHF) significantly improved local control and survival probabilities of stage II and III oesophageal cancer, and fractionation schema (AHF vs conventional fractionation (CF)) was a significant variable on local control in the multivariate analysis (Nishimura et al, 1994, 1997). However, both acute and late toxicities were increased in patients treated with AHF. Thus, AHF may not be feasible or beneficial for all patients with oesophageal carcinomas. It is important to select patients who would benefit from AHF before the start of RT.

Potential doubling time (Tpot) measured by flow cytometry using bromodeoxyuridine (BrdU) or iododeoxyuridine (IdU) has been regarded as a parameter of tumour cell proliferation. Several clinical studies have been performed to reveal the relationship between Tpot and local control or survival rates for patients treated by RT. However, the results of these studies have been variable. Some clinical studies indicated that the Tpot may be a predictor of local control after RT in head and neck cancer (Awwad, 1992; Corvo et al, 1993, 1995; Zackrisson et al, 1997) and in uterine cervical cancer (Tsang et al, 1995; Bolger et al, 1996), while others demonstrated that Tpot had no, or borderline, significance for local control (Begg et al, 1992, 1995; Bourhis et al, 1996).

Recently, immunohistochemical staining of proliferation-related antigens has been applied to the measurement of tumour proliferation. This simple method has become widely prevalent as new antibodies for formalin-fixed human tumours have become commercially available. This technique can be used with archival materials of the tumours, allowing retrospective studies to be performed (Yu et al, 1992; Yu and Filipe, 1993). In addition, immunohistochemical study is superior to BrdU labelling index (LI) in that it is not necessary to administer any agent to patients. Potential prognostic values of proliferating cell nuclear antigen (PCNA) and Ki-67 have been studied extensively for many tumour types (Yu et al, 1992; Yu and Filipe, 1993). PCNA is a 36 kDa acidic nuclear protein and has been recognized as a histologic marker for the G1/S phase in the cell cycle. Ki-67 is a proliferation-associated nuclear antigen and is expressed in all cycling cells except for resting cells in the G0 phase.

The purpose of this study was to clarify the relationship of immunohistochemical indices of PCNA and Ki-67 to local control and survival in patients with oesophageal squamous cell carcinomas treated by definitive RT. In addition, whether these immunohistochemical indices are useful in selecting a fractionation regimen was investigated.

### MATERIALS AND METHODS

The formalin-fixed, paraffin-embedded sections of oesophageal cancers were retrieved from the files of Kyoto University Hospital and its two affiliated hospitals, Shizuoka City Hospital and Kyoto Katsura Hospital. Between 1979 and 1996, 65 patients were identified who fulfilled the following criteria: (1) previously untreated

*Received 14 January 1998*
*Revised 8 July 1998*
*Accepted 6 January 1999*

*Correspondence to:* Y Okuno squamous cell carcinomas of the oesophagus; (2) treated by definitive RT; (3) biopsy materials before RT were available for immunohistochemical analysis. There were 53 males and 12 females, with a median age of 68 years old (range 46–87). Staging was according to the International Union Against Cancer (UICC) classification (1987). There were 12 stage I, 13 stage II, 22 stage III and 18 stage IV oesophageal cancers. Details of the patients and tumour characteristics are shown in Table 1. The clinicopathological parameters of all 65 patients were analysed in relation to immunohistochemical results, although 18 stage IV patients were excluded from the analysis of local control and cause-specific survival. Generally speaking, patients with stage IV disease are not candidates for definitive radiotherapy (RT), and their fields and total doses of radiation are smaller than those for patients with stages I–III. Therefore, patients with stage IV are excluded from the analysis of local control and cause-specific survival.

### Radiation therapy

All of the patients were treated by definitive RT of more than 58 Gy. The details of the radiation methods were described previously (Nishimura et al, 1994). Briefly, AHF for oesophageal cancer was started at Kyoto University Hospital and its affiliated hospitals in 1989. Thereafter, most untreated oesophageal cancers of stages II and III were treated with AHF. Among the 65 patients in this analysis, 18 patients were treated with AHF. Two different schemes were used as twice-a-day (b.i.d.) protocols. Patients were treated with concomitant boosts using b.i.d. irradiation (1.8–1.2 Gy plus 1.1–1.2 Gy) with fractionation interval of 5–6 h. In the other b.i.d. protocol, a large radiation field encompassing the gross clinical disease plus sufficient margin was irradiated b.i.d. at 5- or 6-h intervals with the same fraction size of 1.5 Gy. At a total dose of 39–45 Gy, the radiation field was reduced to the gross clinical disease and a boost was given to the total dose of 63–69 Gy.

All of the patients treated before 1989, and patients who refused AHF after 1989, were treated with CF. Forty-seven patients were treated with CF. For these patients, external RT was given by daily doses of 1.8–2.0 Gy to a total dose of 50–70 Gy. Patients with slight to moderate residual tumours at the end of external RT were selectively treated with intraluminal high-dose-rate brachytherapy (IBT). For these patients, IBT was given once a week following external RT. The reference point of dose calculation was at 5 mm below the surface of the oesophageal mucosa.

Forty-seven patients with disease in stages I–III were divided into two groups according to fractionation schemes. Thirty-two patients were treated with CF, and the remaining 15 patients were treated with AHF. All but one patient with stage I disease were treated with CF. For the CF group, external RT doses ranged from 50 Gy to 70 Gy (mean 62 Gy), and 11 patients (34%) were treated with IBT following external RT. Mean RT dose for IBT was 9.6 Gy by one to four fractions. The patients treated with AHF received external RT doses of 63–69 Gy (mean 66 Gy). Three patients were treated with IBT as boost therapy following AHF. External RT dose for the AHF group was significantly higher than that for the CF group ($P < 0.05$). However, total RT dose (external RT plus IBT) was not significantly different between the two groups. OTT for the external RT of the AHF group ranged from 27 to 49 days with a median of 35 days, while the median of OTT in the CF group was 52 days. Thus, the shortening of OTT by approximately 2 weeks could be achieved by AHF. The difference in OTT between the two groups was significant ($P < 0.003$).

**Table 1** Patients and tumour characteristics

| No. of patients | 65 |
|---|---|
| Sex (male/female) | 53/12 |
| Age (median) | 46–87 (68) |
| Site | |
| Cervical | 5 |
| Upper | 12 |
| Middle | 37 |
| Lower | 11 |
| Histology (differentiation) | |
| Well | 23 |
| Moderate | 34 |
| Poor | 8 |
| Tumour length | |
| < 5 cm | 26 |
| ≥ 5 cm | 39 |
| Stage (UICC, 1987) | |
| I | 12 |
| II | 13 |
| III | 22 |
| IV | 18 |
| T classification | |
| 1 | 12 |
| 2 | 8 |
| 3 | 25 |
| 4 | 20 |
| N classification | |
| 0 | 21 |
| 1 | 39 |
| χ | 5 |
| M classification | |
| 0 | 47 |
| 1 | 18 |

### Chemotherapy

Concomitant chemotherapy was given to two patients in the AHF group, while two patients in the CF group received concomitant chemotherapy. Cisplatin (80–120 mg in total) plus 5-fluorouracil (5-FU) (4000 mg in total) were given during external RT for three patients, and cisplatin alone (200 mg in total) was given to one patient.

### Evaluation of tumour response

Responses of the primary tumour to irradiation were assessed by serial oesophagography, endoscopy and biopsies. Oesophagography and/or endoscopy was performed every 3–4 months for asymptotic patients, and any clinically suspected tumour recurrence required biopsy and histopathological proof. Computerized tomography (CT) scans were obtained at 3- to 6-month intervals for most recent patients, and those were used for evaluation of any recurrence of primary tumours and regional lymph nodes. Local control was defined as neither clinical nor pathological evidence of a primary tumour or lymph nodes recurrence in the initial field of RT.

### Immunohistochemical staining

All specimens were formalin-fixed, paraffin-embedded sections obtained by endoscopic biopsies before RT. The sections were

 *© Cancer Research Campaign 1999*

**Table 2** PCNA labelling index (LI) and clinicopathologic parameters

| | | PCNA LI | | |
|---|---|---|---|---|
| | | **Mean ± s.d.** | **Median** | ***P*-value** |
| No. of patients | 65 | | | |
| Sex | | | | |
| Male | 53 | 50 ± 13 | 52 | NS |
| Female | 12 | 48 ± 13 | 50 | |
| Site | | | | |
| Cervical | 5 | 46 ± 15 | 44 | NS |
| Thoracic | 60 | 50 ± 13 | 53 | |
| Histology (differentiation) | | | | |
| Well | 23 | 51 ± 12 | 54 | NS |
| Moderate | 34 | 48 ± 13 | 51 | |
| Poor | 8 | 52 ± 13 | 53 | |
| Tumour length | | | | |
| $<$ 5 cm | 26 | 50 ± 12 | 52 | NS |
| ≥ 5 cm | 39 | 49 ± 14 | 53 | |
| Stage (UICC, 1987) | | | | |
| I | 12 | 54 ± 7 | 56 | NS |
| II–IV | 53 | 49 ± 14 | 52 | |
| T classification | | | | |
| 1, 2 | 20 | 50 ± 12 | 53 | NS |
| 3, 4 | 45 | 50 ± 13 | 53 | |
| N classification | | | | |
| 0 | 21 | 50 ± 14 | 54 | NS |
| 1 | 39 | 49 ± 12 | 52 | |
| χ | 5 | 54 ± 10 | 55 | |
| M classification | | | | |
| 0 | 48 | 49 ± 13 | 52 | NS |
| 1 | 17 | 51 ± 12 | 53 | |

s.d., standard deviation; NS, not significant.

**Table 3** Ki-67 labelling index (LI) and clinicopathologic parameters

| | | Ki-67 LI | | |
|---|---|---|---|---|
| | | **Mean ± s.d.** | **Median** | ***P*-value** |
| No. of patients | 65 | | | |
| Sex | | | | |
| Male | 53 | 44 ± 16 | 46 | NS |
| Female | 12 | 37 ± 17 | 33 | |
| Site | | | | |
| Cervical | 5 | 26 ± 13 | 22 | $<$ 0.05 |
| Thoracic | 60 | 44 ± 16 | 46 | |
| Histology (differentiation) | | | | |
| Well | 23 | 42 ± 13 | 41 | NS |
| Moderate | 34 | 44 ± 18 | 46 | |
| Poor | 8 | 43 ± 16 | 48 | |
| Tumour length | | | | |
| $<$ 5 cm | 26 | 48 ± 14 | 49 | $<$ 0.05 |
| ≥ 5 cm | 39 | 40 ± 17 | 39 | |
| Stage (UICC, 1987) | | | | |
| I | 12 | 49 ± 9 | 49 | $<$ 0.05 |
| II–IV | 53 | 42 ± 17 | 43 | |
| T classification | | | | |
| 1, 2 | 20 | 49 ± 11 | 49 | $<$ 0.05 |
| 3, 4 | 45 | 40 ± 18 | 40 | |
| N classification | | | | |
| 0 | 21 | 44 ± 16 | 46 | NS |
| 1 | 39 | 43 ± 17 | 45 | |
| χ | 5 | 36 ± 11 | 32 | |
| M classification | | | | |
| 0 | 48 | 42 ± 17 | 46 | NS |
| 1 | 17 | 44 ± 15 | 45 | |

s.d., standard deviation; NS, not significant.

*© Cancer Research Campaign 1999*

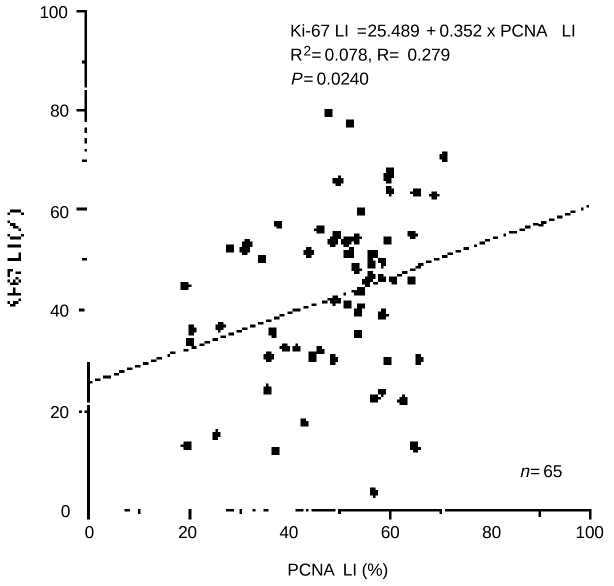

**Figure 1** Relationship between the PCNA LI and the Ki-67 LI. There was a weak but significant correlation between them (R^2 = 0.078, $P$ = 0.0240)

stained by the labelled streptavidin biotin (LSAB) method using a Dako LSAB kit (Dako, Carpinteria, CA, USA). Anti-PCNA mouse monoclonal antibody, PC10 (Cat# NA03, Oncogene Science, Cambridge, MA, USA) and anti-Ki-67 mouse monoclonal antibody, MM1 (NCL–Ki67–MM1, Novocastra Laboratories, UK) were used as the primary antibodies. For retrieval of antigenicity, microwave irradiation in 10 mM sodium citrate buffer at 500 W for 15 min was given for PC10, and autoclave in the target retrieval solution (Dako, Carpinteria, CA, USA) at 121°C for 15 min was processed for MM1. Endogenous peroxide activity was blocked with 3% hydrogen peroxide at room temperature for 10 min. After washing with phosphate-buffered saline (PBS), the sections were incubated with 10% normal swine serum in PBS for 30 min. After that, the sections were incubated with a 1:200 dilution of PC10 at room temperature for 60 min, or with a 1:100 dilution of MM1 overnight at 4°C. The primary antibodies were localized by sequential application of biotylinated anti-mouse IgG gout immunoglobins, streptavidin–peroxide conjugate (Dako, Carpinteria, CA, USA), and diaminobenzidine with 0.03% hydrogen peroxide. The sections were counterstained with haematoxylin and mounted. Control sections were prepared by performing immunohistochemistry using non-immune mouse γ-globulin instead of the primary antibodies.

### Quantitative analysis of immunostaining results

All specimens from the 65 patients were reviewed by a pathologist to confirm the diagnosis and the differentiation. PCNA and Ki-67 LIs were calculated as the percent of PCNA- and Ki-67-positive cancer cells by counting more than 1000 cancer cells of more than three fields of a specimen on × 400 magnification microscopy without knowing any clinical information. Only strong nuclear staining was regarded as positive, and weak nuclear or cytoplasmic staining was regarded as negative.

### Statistical methods

Local control and cause-specific survival were calculated from the first date of external RT. Cause-specific survival considered deaths due only to oesophageal cancer, while all other patients who died of intercurrent diseases were counted as withdrawn alive.

Local control and survival were plotted using the Kaplan–Meier method with statistical significance assessed by the log-rank test. The $\chi^2$ test with Yates correction and Student's $t$-test were used to evaluate the differences in the distribution of pretreatment and treatment parameters.

The Cox proportional hazards model was used to evaluate the significance of prognostic variables on local control. The Cox model was performed using Stat View, version 4.5 (Abacus Concepts, Inc., NC, USA). In the Cox model, 47 patients with stages I–III were included in the analysis of the effect of covariates on local control. The final model considers only those variables that were statistically significant at the 5% level in stepwise regression.

### RESULTS

### Staining of PCNA and Ki-67

Both PCNA and Ki-67 staining were identified in the nuclei of both neoplastic and non-neoplastic cells. The nuclei of the basal and parabasal cells were positive for the markers. In the cancer cells, there were heterogeneous PCNA and Ki-67 staining. In well-differentiated oesophageal cancers, this staining was noted more dominantly in the periphery area of the tumour nest than in the central keratinizing area, while there was a more diffuse staining in poorly differentiated cancers.

The PCNA LI ranged from 20% to 72%, and its mean, median and standard deviation (s.d.) were 50%, 52% and 13% respectively. The relationship of the PCNA LI to clinicopathologic parameters is shown in Table 2. The PCNA LI had no significant relationship to the age or sex of the patients, tumour location, differentiation, tumour length, stage and tumour, node and metastasis (TNM) classification of the lesions. Thus, PCNA LI was independent of known prognostic factors on local control probability for oesophageal cancers.

The Ki-67 LI of the 65 patients ranged from 3% to 79%, and its mean, median and s.d. were 43%, 45% and 16% respectively. The relationship of the Ki-67 LI to clinicopathologic parameters is shown in Table 3. The Ki-67 LI had no significant relationship to the sex of the patients, tumour differentiation, and N- and M-classification. However, the Ki-67 LI was significantly associated with tumour location, clinical stage and T classification. The Ki-67 LI of the cervical oesophageal carcinomas was significantly lower than that of the thoracic lesions ($P < 0.05$). The Ki-67 LI of the patients with tumour lengths of < 5 cm were significantly higher than that of the patients with tumour lengths of ≥ 5 cm ($P < 0.05$). The Ki-67 LI of the patients with T1,2 or stage I disease were significantly higher than that of the patients with T3,4 or stages II–IV diseases (both $P < 0.05$).

To evaluate the relationship between PCNA LI and Ki-67 LI, these LIs were plotted for each tumour (Figure 1). A weak but significant correlation was observed between PCNA LI and Ki-67 LI (R^2 = 0.078, $P$ = 0.024).

### Local control and survival rates according to PCNA LI

For the 47 patients with stage I–III disease, the relationship between the PCNA LI and local control or cause-specific survival rates was analysed. The patients were divided into two groups

 © Cancer Research Campaign 1999

**Table 4** Tumour characteristics according to groups

| Groups | Group A | Difference | Group B | Group C | Difference | Group D |
|---|---|---|---|---|---|---|
| No. of patients | 21 | | 26 | 21 | | 26 |
| Histology (differentiation) | | | | | | |
| Well | 8 | NS | 11 | 9 | NS | 10 |
| Moderate | 11 | | 12 | 10 | | 13 |
| Poor | 2 | | 3 | 2 | | 3 |
| Tumour length | 5.6 ± 2.9 | NS | 5.3 ± 3.2 | 6.2 ± 3.0 | NS | 4.9 ± 2.9 |
| Stage (UICC, 1987) | | | | | | |
| I | 4 | NS | 8 | 2 | NS | 10 |
| II, III | 17 | | 18 | 19 | | 16 |
| T classification | | | | | | |
| 1, 2 | 8 | NS | 11 | 5 | NS | 14 |
| 3, 4 | 13 | | 15 | 16 | | 12 |
| N classification | | | | | | |
| 1 | 10 | NS | 11 | 8 | NS | 13 |
| 0 | 9 | | 12 | 9 | | 12 |
| χ | 2 | | 3 | 4 | | 1 |

Group A: patients with PCNA LI of less than 52%; Group B: patients with PCNA LI of more than or equal to 52%; Group C: patients with Ki-67 LI of less than 45%; Group D: patients with Ki-67 LI of more than or equal to 45% NS, not significant

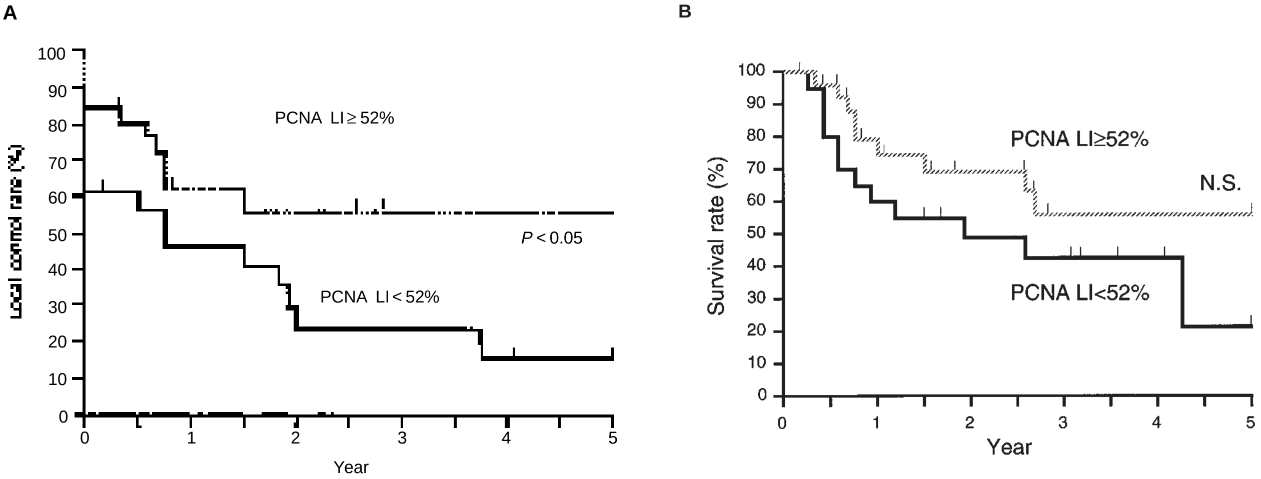

**Figure 2** (**A**) Local control probability and (**B**) cause-specific survival according to PCNA LI. Local control probability of the patients with PCNA LI of ≥ 52% was significantly higher than that of the patients with PCNA LI of < 52% ($P < 0.05$). There was no significant difference in cause-specific survival between the two groups according to the median PCNA LI: group A was the PCNA LI of less than 52% ($n = 21$), and group B was the LI of more than or equal to 52% ($n = 26$). There was no difference in the clinicopathologic parameters between groups A and B (Table 4). Figure 2 shows local control and cause-specific survival of stage I–III patients according to the PCNA LI. The 3-year local control rates for groups A and B were 24% and 56% respectively. The local control probability of group B was significantly higher than that of group A ($P < 0.05$). The 3-year cause-specific survival rates for groups A and B were 43% and 56% respectively. Although the survival rate was better in group B than in group A, the difference was not significant.

### Local control and survival rates according to Ki-67 LI

Similar analysis was performed for the Ki-67 LI. The 47 patients with stage I–III disease were divided into two groups: group C was the Ki-67 LI of less than 45% ($n = 21$) and group D was the LI more than or equal to 45% ($n = 26$). There was no difference in the clinicopathologic parameters between groups C and D (Table 4). Figure 3 shows local control probability and cause-specific survival according to the Ki-67 LI. The 3-year local control probabilities for group C and group D were 21% and 51% respectively. The local control probability of group D was significantly higher than that of group C ($P < 0.05$). The 3-year cause-specific survival

© Cancer Research Campaign 1999

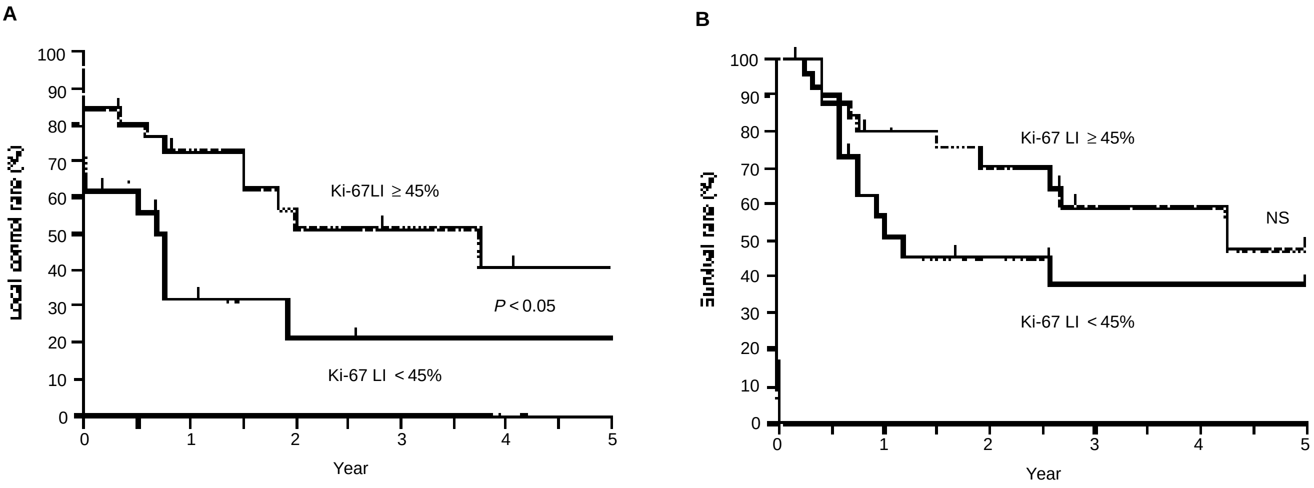

**Figure 3** (**A**) Local control probability and (**B**) cause-specific survival according to Ki-67 LI. Local control probability of the patients with Ki-67 of LI ≥ 45% was significantly higher than that of the patients with Ki-67 LI of < 45% ($P < 0.05$). There was no significant difference in cause-specific survival between the two groups

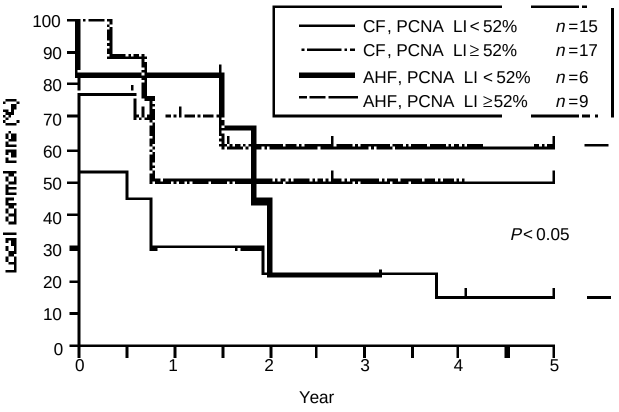

**Figure 4** Relationship between local control probability and the PCNA LI according to fractionation scheme. For the patients treated with conventional fractionation (CF), the local control probability of the tumours with the LI of ≥ 52% were significantly higher than those with the LI of < 52% ($P < 0.05$). No significant difference was observed according to the PCNA LI for patients treated with AHF

for group C and group D were 38% and 60% respectively. There was no difference in the cause-specific survival between groups C and D.

### Local control rate according to fractionation schemes

To assess the impact of the fractionation on local control, the patients were divided into two groups according to fractionation schemes, and the relationship between the LIs and local control probabilities was analysed in each fractionation group. Figure 4 shows the relationship between local control probability and the PCNA LI according to fractionation regimen. For the patients treated with CF, the local control probability of the tumours with high PCNA LI was significantly higher than that with low PCNA LI ($P < 0.05$), although there was no significant difference in local control probability according to the PCNA LI for the patients treated with AHF.

Figure 5 shows the correlation between local control probability and the Ki-67 LI according to the fractionation regimen. For the patients treated with CF, the patients with the high Ki-67 LI had significantly better local control probability than those with the low Ki-67 LI ($P < 0.05$). For the patients treated with AHF, however, there was no significant difference in local control probability according to Ki-67 LI.

### The multivariate analysis

The results of the multivariate analysis using the Cox proportional hazards model are shown in Table 5. The prognostic factors which influenced local control significantly in the univariate analysis were as follows: tumour length (< 5 cm vs ≥ 5 cm; $P < 0.05$), T-stage (T1,2 vs T3,4; $P < 0.005$), N-stage (N0 vs N1; $P < 0.02$), clinical stage (stage I vs stages II–III; $P < 0.05$), PCNA LI (< 52% vs ≥ 52%; $P < 0.05$), and Ki-67 LI (< 45% vs ≥ 45% $P < 0.05$). Because there was significant correlation between T-stage and length, or between clinical stage and length, only T-stage was included in the multivariate analysis among these prognostic factors. N-stage was excluded because N-stages of five patients were unknown because of the lack of the pretreatment chest CT scan. Thus, the following four factors that may have contributed to local control were considered simultaneously in the initial mode of the Cox proportional hazards model. The variables included were T-stage (T1,2 vs T3,4), fractionation schedule (CF vs AHF), PCNA LI (< 52% vs ≥ 52%) and Ki-67 LI (< 45% vs ≥ 45%). The significant factors in the initial model were T-stage ($P = 0.0054$) and PCNA LI ($P = 0.0488$). In the final model using a stepwise regression procedure, T-stage ($P = 0.0056$) and PCNA LI ($P = 0.0332$) were also significant factors for local control. As no significant correlation was found between T-stage and PCNA LI (Table 2), these two variables were independent prognostic factor for local control.

### DISCUSSION

Measurement of Tpot was used as a predictive value of local control or survival in patients treated by RT and to select an appropriate fractionation regimen in head and neck carcinoma (Begg et al, 1990, 1992; Awwad, 1992; Corvo et al, 1993, 1995; Zackrisson et al, 1997) and in uterine cervical cancer (Tsang et al, 1995;

**Table 5** Results of multivariate analysis using Cox proportional hazard model: prognostic variables for local control

| Variables | Initial model (*P*-values) | Final model (stepwise regression) |
|---|---|---|
| Fractionation (AHF vs. CF) | 0.3102 | |
| T stage (T1, 2 vs T3, 4) | 0.0054 | 0.0056 |
| PCNA LI (< 52% vs ≥ 52%) | 0.0488 | 0.0332 |
| Ki-67 LI (< 45% vs ≥ 45%) | 0.3882 | |

AHF, accelerated hyperfractionation; CF, conventional fractionation.

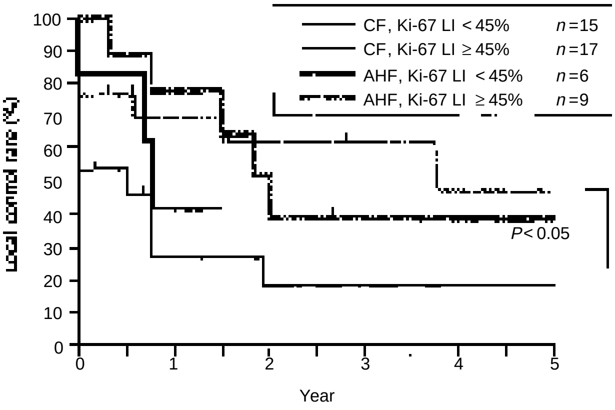

**Figure 5** Relationship between local control probability and the Ki-67 LI according to fractionation scheme. For the patients treated with conventional fractionation (CF), the local control probability of the tumours the LI of ≥ 45% were significantly higher than those with the LI of < 45% ($P < 0.05$). No significant difference was observed according to the PCNA LI for patients treated with AHF

Bolger et al, 1996). In a preliminary analysis of the results of the EORTC trial (Begg et al, 1990), patients with short Tpot tumours had poor local control if given CF. However, in a subsequent analysis, the difference in local control according to the Tpot was not significant (Begg et al, 1992; Begg, 1995). Recently, Zackrisson et al demonstrated that Tpot and nodal status were significant variables on local control in head and neck cancer patients treated by conventional radiotherapy in the multivariate analysis (Zackrisson et al, 1997). Thus, the results of clinical studies on Tpot are still controversial.

PCNA LI and Ki-67 LI have been regarded as parameters of tumour proliferation. In most series of patients undergoing surgery for oesophageal squamous cell carcinomas (Youssef et al, 1995; Kinugasa et al, 1996; Lam et al, 1996), the groups with a high PCNA or a high Ki-67 index showed lower survival rates. For example, Lam et al (1996) analysed PCNA and Ki-67 LIs for surgically resected oesophageal squamous cell carcinoma, and they found that patients with Ki-67 LI of < 30% had better survival rates than those with LI of > 30% in stage III disease. On the other hand, for patients treated mainly by RT, the tumours with high PCNA LI or Ki-67 LI showed good local control in squamous cell carcinomas of the head and neck (Raybaud et al, 1997) and uterine cervical cancer (Oka et al, 1992; Nakano and Oka, 1993; Nakano et al, 1997). In the present study, patients with a PCNA LI of ≥ 52% or those with a Ki-67 LI of ≥ 45% showed a lower local recurrence rate. This result may conflict with the results of Tpot studies. One explanation is that Tpot and LIs of Ki-67 and PCNA represent different aspects of the tumour proliferation. Growth fraction and cell cycle time are major independent tumour proliferation characteristics. Ki-67 and PCNA LIs reflect mainly growth fraction, while Tpot reflects gross proliferation activity including both growth fraction and cell cycle time. Good local control rate for the patients with high PCNA LI or high Ki-67 LI treated by RT may be attributable to a high population of cycling cells. These cycling cells are generally more radiosensitive than quiescent cells (Rodriguez et al, 1988; Mendonca et al, 1989; Masunaga et al, 1991).

AHF has been considered to be more effective for rapidly proliferating tumours. Therefore, initially we expected that local control probabilities of patients with high LIs was poor and AHF may be more effective for tumours with high LIs than for those with low LIs. However, the results obtained contradicted our hypothesis; local control probabilities of patients with low PCNA LI or low Ki-67 LI were poor. Although initial local control rate within 6–12 months of RT was higher in the AHF group than in the CF group for tumours with low LIs (Figures 4 and 5), AHF did not improve the ultimate local control probability significantly for these tumours. Although the number of patients analysed was too small to draw any conclusions, AHF may not be effective for tumours with low LIs or tumours with many quiescent cells. As the present study did not measure the speed of tumour proliferation or Tpot, our results do not imply that AHF is not effective for tumour with rapid proliferation.

In the present study, both PCNA LI and Ki-67 LI had significant correlation with local control probability (both $P < 0.05$) in the univariate analysis, and PCNA LI was also significant for local control in the multivariate analysis. In addition, PCNA LI is superior to Ki-67 LI in that PCNA LI was independent of known prognostic factors on local control probability for oesophageal cancers (Table 2). On the other hand, there was significant difference in Ki-67 LI according to tumour length, clinical stage, and T classification (Table 3). Poor local control probability of the patients with low Ki-67 LI may be associated with a long tumour length and/or advanced T stage (Table 4). Decrease of Ki-67 LI in long and/or advanced tumours may be attributable to reduced proliferative activity and/or loss of Ki-67 antigen expression due to the worsening of the intratumoural environment in the advanced tumours. It is natural that tumour proliferation is influenced by the intratumoural environment. Proliferation is suppressed when either nutrition or oxygen is lacking. For example, Porschen et al (1994) demonstrated that Ki-67 immunostaining of tumour cells decreased with increasing distance from the surrounding capillaries in oesophageal squamous cell carcinomas. Veheijen et al (1989) reported a loss of Ki-67 antigen reactivity in nutritionally deprived cells which were still in S, G2 or M phase as measured by flow

© Cancer Research Campaign 1999

cytometry. Tsurusawa and Fujimoto (1995) reported a down-regulation of Ki-67 antigen expression to an undetectable level in tumour cells with a relatively long G1 duration. In these conditions, Ki-67 LI may be measured lower than the true growth fraction.

In terms of staining technique, Ki-67 LI is superior to PCNA LI. Staining of Ki-67 was easier to interpret than that of PCNA because of less background staining and stronger and more uniform positive stains. The need for caution has been noted when measuring PCNA LI using antibody PC10 on formalin-fixed human tumours (Yu et al, 1992; Coltrera et al, 1993; McCormick et al, 1993*a*; Yu and Filipe, 1993) including: (1) at least two intracellular forms of PCNA exist – a replicon-associated form and a non-associated form that is present in almost all cycling cells (Bravo and Macdonald, 1987); (2) the PCNA staining was found to be highly influenced by the primary antibody dilution and the fixation time, and it lacks a clear plateau (Wolf and Dittrich, 1992; Coltrera et al, 1993; McCormick et al, 1993*b*); (3) the long half-life of PCNA (approximately 20 h), results in stain remaining in cells which have recently left the cell cycle (Bravo and Macdonald, 1987). Therefore, several investigators recommended that Ki-67 LI is a reliable tool for the measurement of proliferation activity (Yu et al, 1992; McCormick et al, 1993*a*; Yu and Filipe, 1993) and is suitable for multi-institute cooperative studies. In those situations, wide variations in fixation times, formaline composition and antibody dilution among different laboratories could result in significant inter-laboratory differences.

In the present study, PC10 was used as the primary antibody for the measurement of the PCNA LI in the formalin-fixed tissues. Therefore, the PCNA LI in this study could include both a replication-associated form and non-associated form. That may be why there was only a weak correlation between PCNA LI and Ki-67 LI (R^2 = 0.078, $P$ = 0.0240). In spite of the technical problem mentioned above, there was a significant correlation between the PCNA LI and local control probabilities ($P < 0.05$) at least by our laboratory method. The characteristics in our method of staining and counting are as follows: (1) microwave irradiation was processed for retrieval of antigenicity, (2) only strong nuclear staining was judged by positive, and weak nuclear or cystoplasmic staining was as negative. Antigen retrieval process may overcome the problem of the various level of loss of antigenicity due to the various fixation times (Greenwell et al, 1991). Strong nuclear staining may reflect a replication-associated from of PCNA (Yu et al, 1995). In the studies where alcohol was used as a fixative, a good correlation between PCNA expression and BrdU labelling indices or the flow cytometrically determined S phase fraction is reported because the replication-associated from of PCNA remains in the cell after alcoholic fixation (Coltrera et al, 1993;Tinnemans et al, 1995). In a prospective study on PCNA staining, alcohol fixation may be better than formalin fixation.

### CONCLUSIONS

In the univariate analysis, PCNA LI and Ki-67 LI of oesophageal squamous cell carcinomas had a significant impact on local control probability of oesophageal cancer treated by definitive RT. PCNA LI was independent of known prognostic variables, and was also a significant variable on local control in the multivariate analysis. For the patients with low PCNA LI or Ki-67 LI, RT by CF regimen was insufficient. For these poor prognostic oesophageal cancers, strong regimens of RT may be necessary, although AHF did not improve significantly the local control probability in the present analysis.

### ACKNOWLEDGEMENTS

The authors wish to thank Dr H Yamabe (Division of Pathology, Kyoto University Hospital), Dr T Itoh (Division of Pathology, Shizuoka City Hospital) and Dr Y Shimada (Division of 1st Surgery, Kyoto University Hospital) for preparation of the specimens and technical suggestions. This study was supported in part by a Grant-in-Aid for Scientific Research from the Ministry of Education, Science, Sports, and Culture, Japan, and a Grant-in-Aid for Cancer Research from the Ministry of Health and Welfare, Japan.

### REFERENCES

Awwad H (1992) Unconventional fractionation studies and Tpot correlation. *Semin Radiat Oncol* **2**: 62–66

Begg AC (1995) The clinical status of Tpot as a predictor? Or why no tempest in the Tpot! [editorial; comment]. *Int J Radiat Oncol Biol Phys* **32**: 1539–1541

Begg AC, Hofland I, Moonen L, Bartelink H, Schraub S, Bontemps P, Le FR, Van DBW, Caspers R, Van GM and Horiot JC (1990) The predictive value of cell kinetic measurements in a European trial of accelerated fractionation in advanced head and neck tumors: an interim report. *Int J Radiat Oncol Biol Phys* **19**: 1449–1453

Begg AC, Hofland I, Van GM and Horiot JC (1992) Predictive value of potential doubling time for radiotherapy of head and neck tumor patients: results from the EORTC cooperative trial 22851. *Semin Radiat Oncol* **1**: 22–25

Bolger BS, Symonds R, Stanton PD, MacLean AB, Burnett R, Kelly P and Cooke TG (1996) Prediction of radiotherapy response of cervical carcinoma through measurement of proliferation rate. *Br J Cancer* **74**: 1223–1226

Bourhis J, Dendale R, Hill C, Bosq J, Janot F, Attal P, Fortin A, Marandas P, Schwaab G, Wibault P, Malaise EP, Bobin S, Luboinski B, Eschwege F and Wilson G (1996) Potential doubling time and clinical outcome in head and neck squamous cell carcinoma treated with 70 Gy in 7 weeks [see comments]. *Int J Radiat Oncol Biol Phys* **35**: 471–476

Bravo R and Macdonald BH (1987) Existence of two populations of cyclin/proliferating cell nuclear antigen during the cell cycle: association with DNA replication sites. *J Cell Biol* **105**: 1549–1554

Coltrera MD, Skelly M and Gown AM (1993) Anti-PCNA antibody PC10 yields unreliable proliferation indexes in routinely processed, deparaffinized, formalin-fixed tissue. *Appl Immunohistochem* **1**: 193–200

Corvo R, Giaretti W, Sanguineti G, Geido E, Orecchia R, Barra S, Margarino G, Bacigalupo A and Vitale V (1993) Potential doubling time in head and neck tumors treated by primary radiotherapy: preliminary evidence for a prognostic significance in local control. *Int J Radiat Oncol Biol Phys* **27**: 1165–1172

Corvo R, Giaretti W, Sanguineti G, Geido E, Orecchia R, Guenzi M, Margarino G, Bacigalupo A, Garaventa G, Barbieri M and Vitale V (1995) In vivo cell kinetics in head and neck squamous cell carcinomas predicts local control and helps guide radiotherapy regimen. *J Clin Oncol* **13**: 1843–1850

Greenwell A, Foley JF and Maronpot RR (1991) An enhancement method for immunohistochemical staining of proliferating cell nuclear antigen in archival rodent tissue. *Cancer Lett* **59**: 251–256

Kinugasa S, Tachibana M, Hishikawa Y, Abe S, Yoshimura H, Monden N, Dhar DK and Nagasue N (1996) Prognostic significance of proliferating cell nuclear antigen (PCNA) in squamous cell carcinoma of the esophagus. *Jpn J Clin Oncol* **26**: 405–410

Lam KY, Law SY, So MK, Fok M, Ma LT and Wong J (1996) Prognostic implication of proliferative markers MIB-1 and PC10 in esophageal squamous cell carcinoma. *Cancer* **77**: 7–13

McCormick D, Chong H, Hobbs C, Datta C and Hall PA (1993*a*) Detection of the Ki-67 antigen in fixed and wax-embedded sections with the monoclonal antibody MIB1. *Histopathology* **22**: 355–360

McCormick D, Yu C, Hobbs C and Hall PA (1993*b*) The relevance of antibody concentration to the immunohistological quantification of cell proliferation-associated antigens. *Histopathology* **22**: 543–547

Masunaga S, Ono K and Abe M (1991) A method for the selective measurement of the radiosensitivity of quiescent cells in solid tumors – combination of immunofluorescence staining to BrdU and micronucleus assay. *Radiat Res* **125**: 243–247

Mendonca MS, Rodriguez A and Alpen EL (1989) Quiescence in 9L cells and correlation with radiosensitivity and PLD repair. *Radiat Res* **117**: 433–447

Nakano T and Oka K (1993) Differential values of Ki-67 index and mitotic index of proliferating cell population. An assessment of cell cycle and prognosis in radiation therapy for cervical cancer. *Cancer* **72**: 2401–2408

© Cancer Research Campaign 1999

Nakano T, Oka K, Ishikawa A and Morita S (1997) Correlation of cervical carcinoma c-erb B-2 oncogene with cell proliferation parameters in patients treated with radiation therapy for cervical carcinoma. *Cancer* **79**: 513–520

Nishimura Y, Ono K, Tsutsui K, Oya N, Okajima K, Hiraoka M and Abe M (1994) Esophageal cancer treated with radiotherapy: impact of total treatment time and fractionation. *Int J Radiat Oncol Biol Phys* **30**: 1099–1105

Nishimura Y, Okuno Y, Hiraoka M, Tsutsui K and Ono K (1997) Acceralated hyperfractionation in the treatment of esophageal squamous cell carcinomas. *Int J Radiat Oncol Biol Phys* **39**: 280

Oka K, Hoshi T and Arai T (1992) Prognostic significance of the PC10 index as a prospective assay for cervical cancer treated with radiation therapy alone. *Cancer* **70**: 1545–1550

Porschen R, Classen S, Piontek M and Borchard F (1994) Vascularization of carcinomas of the esophagus and its correlation with tumor proliferation. *Cancer Res* **54**: 587–591

Raybaud DH, Fortin A, Morency R, Roy J, Monteil RA and Tetu B (1997) Markers of radioresistance in squamous cell carcinomas of the head and neck: a clinicopathologic and immunohistochemical study. *J Clin Oncol* **15**: 1030–1038

Rodriguez A, Alpen EL, Mendonca M and DeGuzman RJ (1988) Recovery from potentially lethal damage and recruitment time of non-cycling clonogenic cells in 9L confluent monolayers and spheroids. *Radiat Res* **114**: 515–527

Tinnemans MM, Lenders MH, Ten VG, Wagenaar SS, Blijham GH, Ramaekers FC and Schutte B (1995) Evaluation of proliferation parameters in in vivo bromodeoxyuridine labelled lung cancers. *Virchows Arch* **427**: 295–301

Trott KR (1990) Cell repopulation and overall treatment time [see comments]. *Int J Radiat Oncol Biol Phys* **19**: 1071–1075

Tsang RW, Fyles AW, Kirkbride P, Levin W, Manchul LA, Milosevic MF, Rawlings GA, Banerjee D, Pintilie M and Wilson GD (1995) Proliferation measurements with flow cytometry Tpot in cancer of the uterine cervix: correlation between two laboratories and preliminary clinical results [see comments]. *Int J Radiat Oncol Biol Phys* **32**: 1319–1329

Tsurusawa M and Fujimoto T (1995) Cell cycle progression and phenotypic modification of Ki67 antigen-negative G1- and G2-phase cells in phorbol ester-treated Molt-4 human leukemia cells. *Cytometry* **20**: 146–153

Verheijen R, Kuijpers HJ, Schlingemann RO, Boehmer AL, van DR, Brakenhoff GJ and Ramaekers FC (1989) Ki-67 detects a nuclear matrix-associated proliferation-related antigen. I. Intracellular localization during interphase. *J Cell Sci*

Withers HR, Taylor JM and Maciejewski B (1988) The hazard of accelerated tumor clonogen repopulation during radiotherapy. *Acta Oncol* **27**: 131–146

Wolf HK and Dittrich KL (1992) Detection of proliferating cell nuclear antigen in diagnostic histopathology. *J Histochem Cytochem* **40**: 1269–1273

Youssef EM, Matsuda T, Takada N, Osugi H, Higashino M, Kinoshita H, Watanabe T, Katsura Y, Wanibuchi H and Fukushima S (1995) Prognostic significance of the MIB-1 proliferation index for patients with squamous cell carcinoma of the esophagus. *Cancer* **76**: 358–366

Yu CC and Filipe MI (1993) Update on proliferation-associated antibodies applicable to formalin-fixed paraffin-embedded tissue and their clinical applications. *Histochem J* **25**: 843–853

Yu CC, Woods AL and Levison DA (1992) The assessment of cellular proliferation by immunohistochemistry: a review of currently available methods and their applications. *Histochem J* **24**: 121–131

Yu CC, Dublin EA, Camplejohn RS and Levison DA (1995) Optimization of immunohistochemical staining of proliferating cells in paraffin sections of breast carcinoma using antibodies to proliferating cell nuclear antigen and the Ki-67 antigen. *Anal Cell Pathol* **9**: 45–52

Zackrisson B, Gustafsson H, Stenling R, Flygare P and Wilson GD (1997) Predictive value of potential doubling time in head and neck cancer patients treated by conventional radiotherapy. *Int J Radiat Oncol Biol Phys* **38**: 677–683

*© Cancer Research Campaign 1999*
